# The Effect of Persuasive Design on the Adoption of Exposure Notification Apps: Quantitative Study Based on COVID Alert

**DOI:** 10.2196/34212

**Published:** 2022-09-06

**Authors:** Kiemute Oyibo, Plinio Pelegrini Morita

**Affiliations:** 1 School of Public Health Sciences Faculty of Health University of Waterloo Waterloo, ON Canada; 2 Department of Electrical Engineering and Computer Science York University Toronto, ON Canada; 3 Institute of Health Policy, Management, and Evaluation University of Toronto Toronto, ON Canada; 4 Department of Systems Design Engineering University of Waterloo Waterloo, ON Canada; 5 eHealth Innovation Techna Institute University Health Network Toronto, ON Canada

**Keywords:** contact tracing app, exposure notification app, COVID Alert, COVID-19, persuasive technology, behavior change, exposure, behavior, effect, design, adoption, use, case study, effectiveness, user interface, mobile phone

## Abstract

**Background:**

The adoption of contact tracing apps worldwide has been low. Although considerable research has been conducted on technology acceptance, little has been done to show the benefit of incorporating persuasive principles.

**Objective:**

This research aimed to investigate the effect of persuasive features in the COVID Alert app, created by Health Canada, by focusing on the no-exposure status, exposure status, and diagnosis report interfaces.

**Methods:**

We conducted a study among 181 Canadian residents, including 65 adopters and 116 nonadopters. This study was based on screenshots of the 3 interfaces, of which each comprised a persuasive design and a control design. The persuasive versions of the first two interfaces supported self-monitoring (of exposure levels), and that of the third interface supported social learning (about how many other users have reported their diagnosis). The 6 screenshots were randomly assigned to 6 groups of participants to provide feedback on perceived persuasiveness and adoption willingness.

**Results:**

A multivariate repeated-measure ANOVA showed that there is an interaction among interface, app design, and adoption status regarding the perceived persuasiveness of the interfaces. This resulted in a 2-way ANOVA for each interface. For the no-exposure interface, there was an interaction between adoption status and app design. Among adopters, there was no significant difference *P*=.31 between the persuasive design (mean 5.36, SD 1.63) and the control design (mean 5.87, SD 1.20). However, among nonadopters, there was an effect of app design (*P<*.001), with participants being more motivated by the persuasive design (mean 5.37, SD 1.30) than by the control design (mean 4.57, SD 1.19). For the exposure interface, adoption status had a main effect (*P<*.001), with adopters (mean 5.91, SD 1.01) being more motivated by the designs than nonadopters (mean 4.96, SD 1.43). For the diagnosis report interface, there was an interaction between adoption status and app design. Among nonadopters, there was no significant difference *P*=.99 between the persuasive design (mean 4.61, SD 1.84) and the control design (mean 4.77, SD 1.21). However, among adopters, there was an effect of app design (*P=*.006), with participants being more likely to report their diagnosis using the persuasive design (mean 6.00, SD 0.97) than using the control design (mean 5.03, SD 1.22). Finally, with regard to willingness to download the app, pairwise comparisons showed that nonadopters were more likely to adopt the app after viewing the persuasive version of the no-exposure interface (13/21, 62% said yes) and the diagnosis report interface (12/17, 71% said yes) than after viewing the control versions (3/17, 18% and 7/16, 44%, respectively, said yes).

**Conclusions:**

Exposure notification apps are more likely to be effective if equipped with persuasive features. Incorporating self-monitoring into the no-exposure status interface and social learning into the diagnosis report interface can increase adoption by >30%.

## Introduction

### Background

The COVID-19 pandemic resulted in the imposition of public health restrictions and the shutting down of several economies by most national governments worldwide. This necessitated the rollout of digital contact tracing apps to curb the spread of the coronavirus. Digital contact tracing apps help notify users who may have come in contact with someone with COVID-19 so that appropriate safety measures such as self-isolation and testing for COVID-19 can be taken [1]. They were mostly rolled out in high-income countries to support manual methods of contact tracing, which are often labor-intensive, time-consuming, and less likely to be accurate because of the limitation of human memories in recalling contacts [2]. They have the potential to reach a critical mass of adopters and are hence more likely to be effective than traditional means of contact tracing. The emergence of new variants of COVID-19 such as Delta variant [3], which may be resistant to vaccines [4], and its endemic potential are an indication that contact tracing apps may continue to be relevant in the fight against COVID-19 in the long term [5,6]. However, their adoption has been very low and slow owing to several factors [7].

Apart from trust- and privacy-related concerns, the minimalist design of contact tracing apps currently on the Google and Apple app stores tends to limit their perceived usefulness [8]. As noted by Kukuk [9], *“*[a]part from providing receiving notifications about possible infections, current contract tracing apps appear to not provide a clear benefit to the user.” Digital health experts have identified the lack of persuasive design and motivational affordances as being partly responsible for the low acceptance of contact tracing apps worldwide [7,10]. Research has shown that 56% of the population (eg, in a given country) may have to use contact tracing apps to considerably slow the spread of the virus [11]. Hence, there is a need for researchers to investigate ways to improve the design of contact tracing apps and increase their effectiveness. The minimalist design of contact tracing apps [8,12] (eg, users not being able to track the number of contacts and exposure time) might have been occasioned by the need to minimize collected user data to reduce privacy concerns [13,14] and eliminate fear of government surveillance [15]. Although, this can be seen as an advantage, it has also reduced the usefulness of contact tracing apps [9]. Research has shown that some users may be willing to provide more of their data to contact tracing apps (eg, location data) to receive additional benefits, such as the ability to track the number of daily contacts they had and COVID-19 hot spots [16,17]. The willingness of some users to provide more user data than others to have access to more useful features is an indication of the need for contact tracing apps tailored to different target groups [10,18].

### Persuasive Design

We argued that the incorporation of persuasive features such as self-monitoring, social learning, tailoring, personalization, expertise, praise, and reward has the potential to improve the perceived persuasiveness of contact tracing apps and the reporting of COVID-19 diagnoses [18]. However, there is limited research on the effectiveness of the persuasive design of contact tracing apps in motivating behavior change. Most prior studies [19-21] did not focus on incorporating persuasive features in contact tracing apps. Rather, they focused on the Technology Acceptance Model (TAM), which does not consider persuasive design attributes. From the viewpoint of the TAM, we argue that the perceived usefulness of existing contact tracing and exposure notification apps through persuasive design has been relegated to the background [9,10]. One plausible explanation for this oversight was the need to roll out contact tracing apps as soon as possible to help flatten the curve.

To bridge the gaps in the extant literature, we proposed design guidelines for incorporating persuasive features in exposure notification apps (see our conceptual paper [18]). The guidelines were drawn from the persuasive system design (PSD) model by Oinas-Kekkonen and Harjumaa [22], which is commonly used in designing, implementing, and evaluating persuasive systems [23,24]. In this study, we implemented and evaluated the perceived persuasiveness of 2 of the proposed persuasive features (self-monitoring and social learning) from our conceptual paper [18], using the Government of Canada’s COVID Alert app as proof of concept [25]. The app was created by Health Canada in collaboration with Blackberry that provided privacy and security guidance [26]. We chose only 2 persuasive strategies because we could not implement and evaluate all persuasive strategies in the PSD model at the same time, and we had to start from somewhere. In particular, we chose self-monitoring because prior work, such as that by Cruz et al [17], reported that contact tracing app users would like to know the number of persons they have come in contact with. Second, we chose social learning because we believed that learning about the number of other users in your community who have reported their COVID-19 diagnosis holds the potential to motivate users to report theirs when they test positive. Moreover, prior research on persuasive technology has demonstrated that social learning has the capacity to motivate people to engage in beneficial behaviors regardless of culture, gender, or age [27,28]. The rationale for choosing self-monitoring and social learning is discussed in further detail in our prior conceptual paper, which focused on designing exposure notification apps as persuasive technologies [18].

### Study Description

We conducted a survey on Amazon Mechanical Turk among 204 participants residing in Canada to investigate the effect of persuasive design on the adoption and perceived persuasiveness of COVID Alert. This study was based on 2 sets of app designs (persuasive and control), 3 types of use cases (no-exposure status interface, exposure status interface, and diagnosis report interface), and 2 types of participants (COVID Alert adopters and nonadopters). The persuasive design supports persuasive features, such as self-monitoring and social learning, whereas the control design does not support any persuasive features. Self-monitoring, which is incorporated into the no-exposure and exposure status interfaces of the COVID Alert app, is one of the most commonly used and effective persuasive strategies in behavior change [29-31]. It provides users with opportunities for self-reflection and self-regulation, which result in increased focus and commitment to achieving a target behavior such as social distancing. Moreover, social learning, which is integrated into the diagnosis report interface, is an effective persuasive strategy for motivating behavior change through social influence and pressure [32]. To evaluate the effectiveness of persuasive design, we carried out a 4-factor multivariate repeated-measure ANOVA (RM-ANOVA) [33] based on interface, app design, adoption status, and perceived persuasiveness. Our overall hypothesis is that the persuasive design of exposure notification apps, regardless of the use case (interface), is more likely to be persuasive and adopted by potential users than the control design. Moreover, we hypothesize that adopters are more likely to find exposure notification apps persuasive than nonadopters, regardless of app design and use case.

### Related Work

#### Overview

Before conducting this research, we searched 6 databases (Scopus, CINAHL, PubMed [MEDLINE], IEEE Xplore Digital Library, ACM Digital Library, and Web of Science) between October 30, 2020, and November 20, 2020, using the following terms: (*contact tracing* OR *contact-tracing* OR *exposure notification* OR *exposure-notification* OR *contact notification* OR *contact-notification* OR *GAEN*) AND (*app* OR *apps* OR application* OR technolog* OR *system* OR *systems*) AND (percept* OR adopt* OR accept* OR *uptake* OR *use* OR *usage*) AND (covid* OR *coronavirus* OR *SARS-CoV-2*). In addition, we searched Google Scholar between November 21, 2020, and January 31, 2021, using terms such as *COVID-19 contact tracing app* and *COVID-19 exposure notification app*. The systematic review, which uncovered the key factors that drive the acceptance of contact tracing apps, is published in Frontiers in Digital Health [34]. The protocol for this review was published in the Journal of Medical Internet Research [35]. In this study, we review the key related articles retrieved from the database search, focusing on privacy, trust, and persuasive design.

#### Privacy and Trust

Privacy and trust are among the top-ranking ethical issues that COVID-19 stakeholders such as researchers, designers, and the public are concerned with when it comes to digital contact tracing [36-38]. In the context of web-based systems, privacy refers to the level of protection and security of user data and interaction while using an electronic system connected to the internet. It entails the collection, storage, use, and sharing of a user’s personal information [39]. In contrast, trust (despite not having a universally accepted scholarly definition [40]), in the context of web-based activities, is regarded as a cognitive mechanism adopted by users when interacting with internet-connected systems. Usually connected to the perceived quality, usability, and expertise of a web-based system such as a website, trust “operates to reduce the amount of [perceived] risk by reducing perceptions of anxiety and uncertainty” [40]. Preliminary research shows that there is a significant relationship between privacy concerns and trust, with each having the potential to impact the adoption of web-based systems, such as social networking sites [41,42] and e-commerce sites [43,44]. For example, Zlatolas et al [41] found that the higher the perceived privacy risk of using Facebook, the lower the perceived trust of users, and the lower the perceived trust in a social media site, the higher the privacy concerns of users. Trust is often associated with the success or failure of an e-commerce website, as web-based shoppers are concerned with unsafe products, insecure payment methods, loss of privacy, identity theft, and misuse of personal information [45].

In the contact tracing domain, research has also shown that privacy concerns and trust can impact the adoption of contact tracing apps [38]. For example, Sharma et al [19], Altmann et al [21], Kaspar [46], and Velicia-Martin et al [47] found in their work on technology acceptance that the higher people’s concern about privacy is, the less likely they are to download, install, or use contact tracing apps. Moreover, Sharma et al [19], Altmann et al [21], and Kaspar [46] found that the higher the users’ perceived trust in contact tracing apps and their stakeholders, such as the government, the higher their likelihood of adopting them. In contrast, Jonker et al [48] and Thomas et al [49] found that the higher the distrust of users (eg, in governments and tech companies [50]), the less likely they are to adopt contact tracing apps. Hence, as a way of enacting privacy protection, Jonker et al [48] recommended that governments implement contact tracing apps with adequate realistic privacy-preserving features; for example, users should be given control over their data, including deciding what data they want to share, whom they want to share it with, how and when they want to share it, and what it will be used for. Similarly, Walrave et al [20] recommended that contact tracing app sponsors inform potential users about the data to be collected and minimize data collection and the amount of time required to read and evaluate privacy terms by using visual presentation to improve comprehension. Finally, in furthering and fostering public trust, Altmann et al [21] recommended that national governments around the world should consider delegating the mandate of digital contact tracing to reputable and transparent public health institutions, over which they have little to no control.

#### Persuasive Design

Although a substantial amount of work has been done with regard to the impact of privacy and trust on contact tracing app adoption (as shown in the previous subsection), little has been done with regard to the impact of persuasive design. As of the time of writing this paper, we found only 2 studies [17,48] that investigated the benefit of incorporating persuasive features in contact tracing apps. One of the studies (Cruz et al [17]) found that more than half of the participants wanted to know how many infected people they had come in contact with (including the location and time) by way of self-monitoring. The study also found that most participants were more willing to share their locations when they were offered tangible rewards [17]. Similarly, another study (Jonker et al [48]) found that participants preferred contact tracing apps that offer tangible rewards, such as money and free COVID-19 testing. However, these studies were primarily based on contact tracing app descriptions and not implementations. Moreover, these studies were not based on a comparative analysis of intervention designs (equipped with persuasive strategies) or control designs (unequipped with persuasive strategies). Most importantly, the studies were carried out in the first half of 2020, when many people were less familiar with or had not used contact tracing and exposure notification apps. Hence, there is a need for this study to bridge the gap in the extant literature regarding the effect of persuasive design on contact tracing and exposure notification app design.

## Methods

In this section, we focus on app design, measurement instruments, recruitment of participants, experimental design and data analysis, sample size calculation, and research model and hypotheses.

### App Design

**Figure 1:**
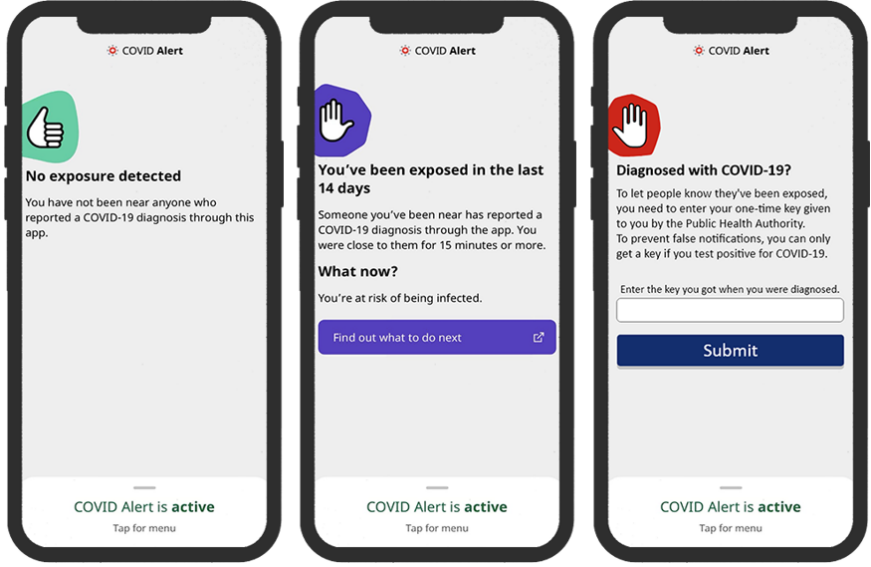
Control designs of the 3 key interfaces of the COVID Alert app.

**Figure 2:**
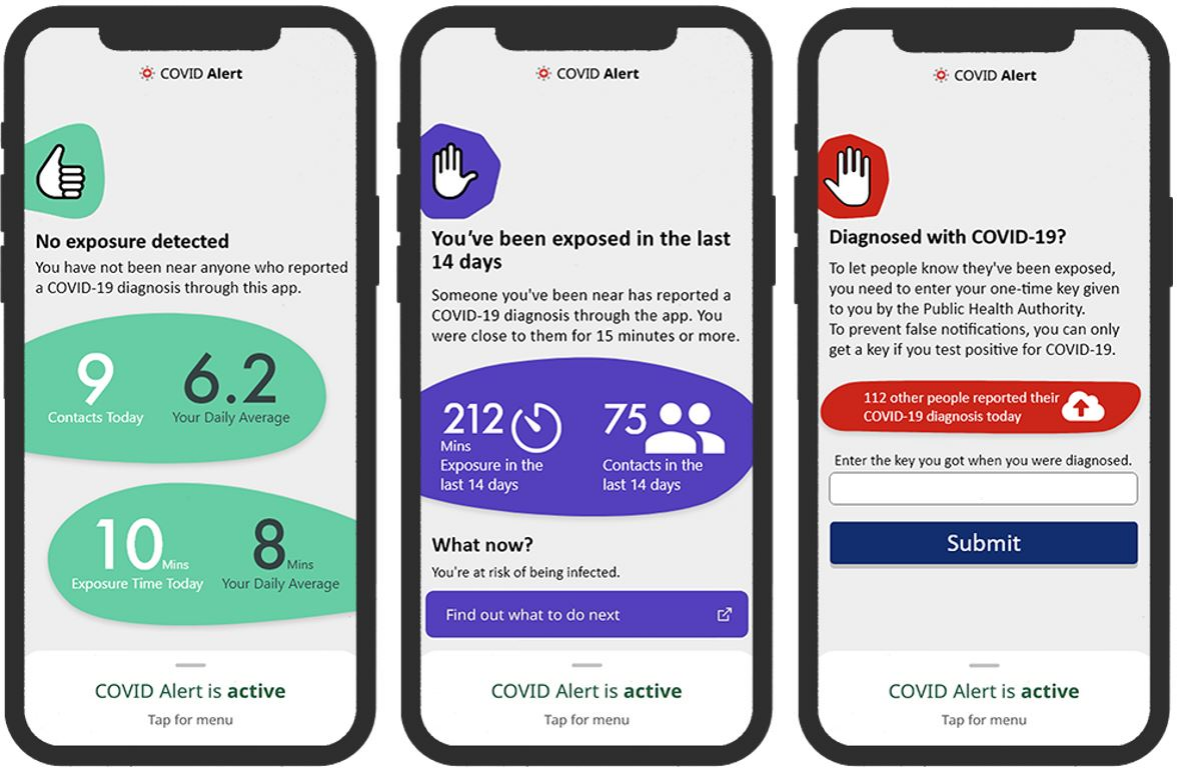
Persuasive designs of the 3 key interfaces of the COVID Alert app.

COVID Alert is the Government of Canada’s official app for contact tracing and exposure notification. Released on July 31, 2020, it uses Google/Apple Exposure Notification application programming interfaces to enforce strong privacy measures. Hence, it does not track the user’s location or collect personally identifiable information such as name, contacts, address, or health information. Similar to many exposure notification apps on the market, the COVID Alert app (persuasive or control design) comprises 3 key use cases: no-exposure status interface, exposure status interface, and diagnosis report interface (Figures 1 and 2). In the persuasive design, we implemented 2 types of persuasive strategies (self-monitoring and social learning) drawn from the PSD model [22]. The PSD model is a framework for the design, implementation, and evaluation of persuasive systems. It comprises 28 persuasive strategies. In our conceptual paper on exposure notification app design [18], we discuss likely persuasive strategies from the PSD model that can be incorporated into exposure notification apps to make them more effective and appealing. These include self-monitoring, tailoring, social learning, normative influence, trustworthiness, and authority. The rationale for implementing these strategies is described in the conceptual paper. In this study, we implemented the aforementioned strategies by focusing on self-monitoring (incorporated into the no-exposure and exposure status interfaces) and social learning (incorporated into the diagnosis report interface).

As shown in Figure 1, the no-exposure status interface informs the user that they have not been exposed to COVID-19 by being close to someone with COVID-19 in the last 14 days. The exposure status interface notifies the user that they may have been exposed to COVID-19 by being in close contact with someone with COVID-19, and provides information on what to do next (eg, self-isolate or go test for COVID-19 in the event of having symptoms). Finally, the diagnosis report interface enables a user who has tested positive to enter a one-time key given to them by the public health authority. We regard these 3 key original interfaces of the COVID Alert app, which are not equipped with persuasive features, as control designs (Figure 1).

**Figure 3:**
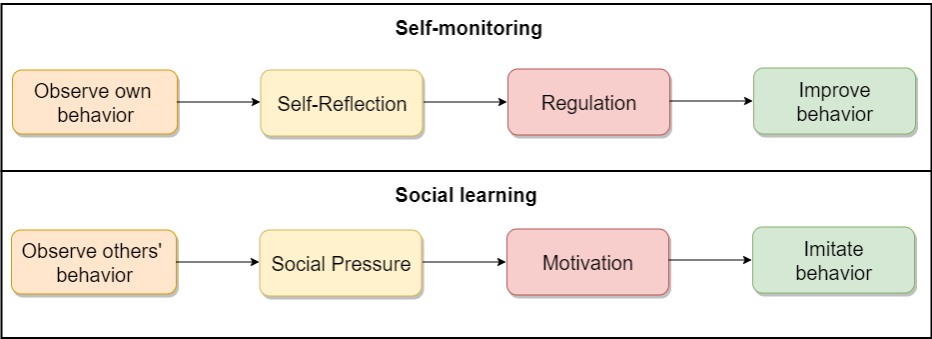
The operational mechanism of self-monitoring and social learning [34,51,52].

Figure 2 shows the corresponding persuasive designs equipped with persuasive features. The no-exposure and exposure status interfaces are equipped with self-monitoring, and the diagnosis report interface is equipped with social learning. Self-monitoring is a persuasive feature that allows users to track their COVID-19 exposure levels over time. Figure 3 [34,51,52] illustrates the operational mechanism of self-monitoring. A person observes their own behavior and reflects on it, as though they are looking at themselves in the mirror. If they are not impressed with what they see (in the mirror), they regulate themselves by improving on the target behavior [29,53,54]. In the no-exposure status interface, users can track total and average number of daily contacts and minutes exposed. In the exposure status interface, users can view the cumulative sum of contacts and exposure minutes in the last 14 days within which they must have been exposed. It is hoped that by seeing these summary statistics, users will be motivated to regulate their social distancing behavior. In contrast, social learning is a persuasive feature that allows users to be aware of other people’s behavior in the hope that they will be socially pressured and motivated to adopt the observed behavior. Figure 3 illustrates the operational mechanisms of social learning [53,55,56]. Social learning is based on the premise that observational learning cannot occur unless cognitive processes that mediate the learning process occur [52]. Figure 3 demonstrates that by observing others' behavior, one is motivated through social pressure to imitate the observe behavior for the common good. In the diagnosis report interface, the app informs the user about the number of users who have reported their COVID-19 diagnosis on a given day in the hope that they would be socially pressured to report if they tested positive to promote public health safety.

### Measurement Instruments

To investigate the effectiveness of the persuasive design, we measured 2 key constructs of interest: perceived persuasiveness of each of the interfaces (shown in Figures 1 and 2) and participants’ willingness to download the COVID Alert app from the app store. Table 1 shows the measures for both the constructs. Perceived persuasiveness refers to and measures the ability of the visual and informational design of an app to motivate users to adopt it. In this study, perceived persuasiveness is a reflective measure that captures how well the visual design of the COVID Alert app convinces and influences the user to start or continue using the app.

**Table 1:** Measurement instruments.

Construct	Items measuring construct
Perceived persuasiveness (“strongly disagree: 1” to “strongly agree: 7”) [57]	The app design (name of interface)... …influences me to start or continue using the COVID Alert app. …is convincing for me to start or continue using the COVID Alert app. …is relevant to my using or continued use of the COVID Alert app.
Willingness to download app from store (yes or no)	Now that I know about the COVID Alert app as the Government of Canada’s official exposure notification app, I will download it from the Apple or Google store to slow down the spread of the coronavirus.
Adoption status	Which of the following best describes you? I am currently using the COVID Alert app. I am currently using a COVID-19 contact tracing or exposure notification app other than COVID Alert. I am not currently using any COVID-19 contact tracing or exposure notification app.

In the context of this study, perceived persuasiveness can be viewed as a proxy for the TAM or Theory of Planned Behavior constructs such as perceived usefulness [56,58], perceived compatibility with existing experiences, values, and tasks [59,60], and peer or superior influence [61], which have the potential to impact the adoption of new technologies. For example, the more a new technology is perceived as useful and compatible with the user’s past experiences, values, and tasks, the more relevant they will deem it and the more likely they will be to adopt it [61]. However, although perceived persuasiveness may be associated with constructs such as perceived ease of use and perceived usefulness [57,58], perceived compatibility with tasks [59], and social influence [62], it is not synonymous with any of these constructs. For example, the fact that a user perceives an app to be persuasive (motivating) may not mean that they find it easy to use, useful, or compatible with prior experiences, values, and tasks or vice versa. One plausible explanation is that some users may perceive an app (eg, a game) to be persuasive based on hedonic characteristics (such as perceived aesthetics [63] and perceived enjoyment [64]), without considering the utilitarian (eg, perceived usefulness) or compatibility features. In contrast, other users may perceive an app (eg, an exposure notification app) to be persuasive based on utilitarian or compatibility features without paying much attention to hedonic features. In the context of the PSD model, perceived persuasiveness can be viewed as a proxy for the four main categories of persuasive strategies. They include primary task support, dialog support, social support, and credibility support, which have direct and indirect relationships with perceived persuasiveness and adoption intention, respectively [65]. In particular, primary task support (defined as persuasive features that enable users to realize the main goal of a persuasive system) can be compared to perceived usefulness in the TAM. For example, in the work by Lehto et al [65], based on a web-based persuasive health system, primary task support was operationalized using utility-oriented items including (1) the system provides me with means to lose weight, (2) the system helps me lose weight, and (3) the system helps me change my eating habits, which reflect perceived usefulness.

For this study, the perceived persuasiveness measure was adapted from the work by Lehto et al [65], to suit the context of exposure notification apps. It is a 7-point scale ranging from *strongly disagree (1)* to *strongly agree (7)*. Moreover, willingness to download refers to and measures participants’ intention to adopt the app to curb the spread of the coronavirus after seeing or learning about its functionality. It was based on a *yes-or-no* measure. Finally, we measured adoption status by asking participants to choose 1 of the 3 options shown in Table 1. If they chose the first and third options, they were regarded as COVID Alert adopters and nonadopters, respectively. Those who chose the second option were filtered out of the data analysis, as we were interested in analyzing and comparing participants who had installed and interacted with the COVID Alert app and those who had not in the past.

### Participants

The criterion for inclusion in the study was that participants must be residents of Canada, regardless of sex, gender, age, education, country of origin, and contact tracing app adoption status. We did not place any demographic restrictions on who could participate in the study because everyone, regardless of the enumerated demographic variables, is liable to be exposed to COVID-19, and is thus expected to use exposure notification apps such as COVID Alert. We recruited participants residing in Canada with at least one year of smartphone use experience on Amazon Mechanical Turk to evaluate the persuasive and control designs of the COVID Alert app. Amazon Mechanical Turk is an inexpensive crowdsourcing web-based commercial platform for recruiting a nonconvenience sample of participants worldwide. Research has shown that owing to its quality-assurance mechanism, the platform has the potential to yield high-quality data [66]. The recruitment of study participants took place between December 25, 2020, and January 25, 2021. With the aid of our laboratory-wide account, the first author used the requester interface to post details of the study on the Amazon Mechanical Turk platform. The requester interface allows the researcher to specify the number of participants, duration of the study, and types of participants using filtering terms such as country and location [67]. We tweaked the default JavaScript code in the requester interface to randomly assign 1 of the 6 exposure notification app interfaces to each potential anonymous participant. Hence, each participant only viewed the interface assigned to them as described in Multimedia Appendix 1, without interacting with it. Before completing the web-based questionnaire, each participant was requested to read the information and consent forms and provide informed consent. Upon consent, participants were allowed to complete the survey; otherwise, they were directed to the end of the survey. Each participant was remunerated with US $2 in appreciation of their time.

A total of 204 participants took part in the study. Of these, 65 (32%) had already used the COVID Alert app, 17 (8%) were using other contact tracing apps, 116 (57%) did not use the COVID Alert app or any other contact tracing app at the time of taking the survey, and 6 (3%) did not specify their adoption status. The first and third subgroups were regarded as the COVID Alert adopter group (n=65) and the nonadopter group (n=116), respectively. The second and fourth subgroups (n=23) were filtered out during data analysis. Table 2 shows the demographics of the COVID Alert adopters and nonadopters (n=181) assigned to the 6 user interfaces, comprising 3 control designs (C1, C2, and C3) and 3 persuasive designs (P1, P2, and P3).

**Table 2:** Participants’ demographics based on the 6 user interfaces (N=181).

Criterion and subgroup		Overall users, n	No-exposure interface, n		Exposure interface, n		Diagnosis report interface, n	
			C1^a^	P1^b^	C2	P2	C3	P3
**Gender**								
	Male	106	16	20	18	21	19	12
	Female	73	10	12	12	16	9	14
	Others	2	1	0	0	0	1	0
**Age** **(years)**								
	<18	1	0	1	0	0	0	0
	18 to 24	36	1	6	7	10	5	7
	25 to 34	64	8	10	12	12	11	11
	35 to 44	48	9	9	6	9	10	5
	45 to 54	19	6	2	3	4	2	2
	>55	10	2	3	1	2	1	1
	Unspecified	3	1	1	1	0	0	0
**Education**								
	Technical or trade	5	0	0	1	2	1	1
	High school	39	2	11	4	9	3	10
	Bachelor’s	99	20	14	18	19	18	10
	Master’s	29	3	4	6	6	5	5
	Doctorate	3	1	1	0	0	1	0
	Other	6	1	2	1	1	1	0
**Using smartphone (years)**								
	1 to 5	27	3	6	2	4	5	7
	6 to 10	86	14	16	18	19	9	10
	11 to 20	59	8	9	10	12	13	7
	>20	8	2	1	0	2	2	1
	Unspecified	1	0	0	0	0	0	1
**Country of origin**								
	Canada	143	24	21	24	31	21	22
	Other	38	3	11	6	6	8	4
**Adoption status**								
	Adopters	65	10	11	11	11	13	9
	Nonadopters	116	17	21	19	26	16	17

^a^C: control design.

^b^P: persuasive design.

### Experimental Design and Data Analysis

This study was based on a web-based questionnaire in which each participant was randomly assigned to 1 of the 6 user interfaces shown in Figures 1 and 2. Before questions were asked to the participants, the functionality of the COVID Alert app was described to them (see Multimedia Appendix 1 for details on the experimental design and accompanying information presented to participants). Two types of data analysis were carried out: path modeling and multivariate RM-ANOVA [33]. First, the path modeling set out to uncover the strength of the relationship between the perceived persuasiveness of each of the 3 interfaces (no-exposure status, exposure status, and diagnosis report) and the willingness to download the app by nonadopters. This analysis helped us establish that there is a significant relationship between the perceived persuasiveness of an exposure notification app and the willingness to adopt it by nonadopters.

Second, the experimental design, based on a 4-way multivariate RM-ANOVA factorial design, aimed to understand the main effect of *app design*, *interface*, and *adoption status* on the *perceived persuasiveness* of each user interface and their interactions. On the basis of this 4-way multivariate RM-ANOVA factorial design, we aimed to understand the main effect of the first 3 variables on the perceived persuasiveness of each of the 3 user interfaces and their interactions. The app design has 2 conditions (*persuasive* and *control*), the interface has 3 levels (*no-exposure status*, *exposure status*, and *diagnosis report*), and the adoption status has 2 levels (*adopters* and *nonadopters*). Moreover, perceived persuasiveness was measured repeatedly using 3 indicators as shown in Table 1. Finally, among the nonadopter group, we investigated the effect of app design on participants’ willingness to download the COVID Alert app from the app store. Using 2×2 chi-square tests [68], we compared, for each user interface, the percentage of participants who viewed the persuasive design that said “yes” with the percentage of participants who viewed the control design that said "yes". This pairwise comparison helped to uncover any significant difference between the persuasive and control design groups.

### Sample Size Calculation

Before conducting this study, we computed the sample size using the University of British Columbia’s web-based power and sample size calculator developed by Brant [69]. We chose the default significance level of .05 and a power level of 0.80. Moreover, we chose our SD value to be 1.0, and the mean difference between the 2 groups as 0.8 on a 7-point Likert scale (ie, >10% difference). The SD was derived from a similar study of the principles of persuasion by Cialdini, conducted among individualist participants from North America [70]. In particular, the SD for the liking principle, which is highly related to the perceived persuasiveness construct in this study, was 1.09. Hence, we decided to use a SD of approximately 1.0 for the calculation of our sample size for each group. The calculation (based on a 2-sided test) resulted in a sample size of 25 for each group. As shown in Table 2, a total of 6 groups met this sample size requirement, with 5 of them being >30.

### Research Model and Hypotheses

**Figure 4:**
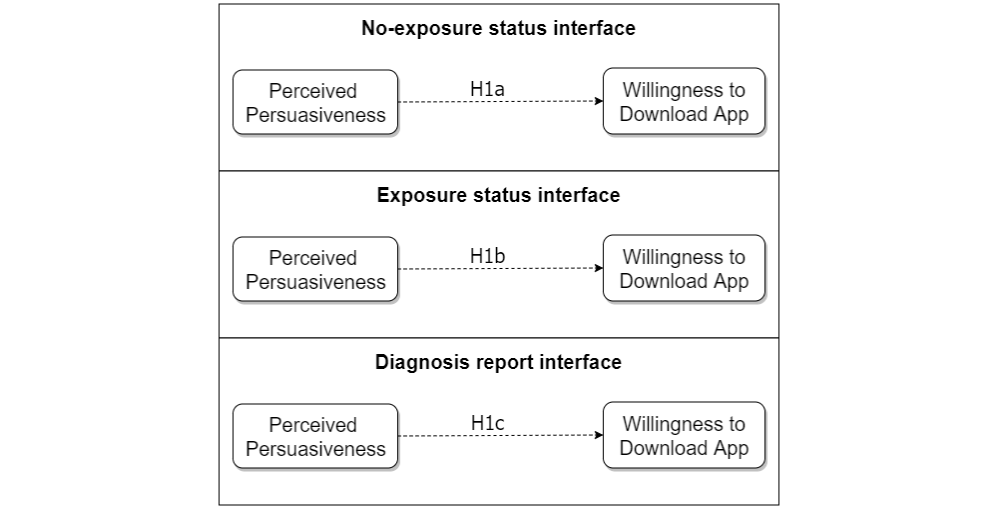
Research model for the relationship between perceived persuasiveness and willingness to download the app by nonadopters. H: hypothesis.

We based our data analysis on path modeling and multivariate RM-ANOVA. Figure 4 shows the hypothesized model. This model was based on prior research, which showed that there is a significantly strong relationship between the perceived persuasiveness of an app (such as a fitness app) and adoption intentions [57]. On the basis of this finding and the fact that screenshots of key interfaces of an app are often included in its description in the app store, we hypothesized as follows: *hypothesis H1: the higher the perceived persuasiveness of an exposure notification app in the app store, the more likely users will download it.* This hypothesis is based on the premise that potential users will be able to view the key interfaces of the app (in addition to reading its description) in the app store before making their decision to download it. It is broken down for each of the 3 key user interfaces as follows:

1. H1a: the higher the perceived persuasiveness of the no-exposure status interface in the app store, the more likely users will download the COVID Alert app.
2. H1b: the higher the perceived persuasiveness of the exposure status interface in the app store, the more likely users will download the COVID Alert app.
3. H1c: the higher the perceived persuasiveness of the diagnosis report interface in the app store, the more likely users will download the COVID Alert app.

In addition, using an exploratory approach, we investigated which of the 3 interfaces (ie, perceived persuasiveness) has the strongest effect on users’ willingness to download the COVID Alert app. It is noteworthy that we do not imply or mean a causal-effect relationship in H1 or each time we use the word *effect* in characterizing the relationship between perceived persuasiveness and willingness to download the app. As the mantra goes, *correlation does not mean causation*. Moreover, we hypothesized that the perceived persuasiveness of each interface will be influenced by the app design. In other words, given that persuasive designs support persuasive features such as self-monitoring and social learning, we hypothesized as follows:

1. H2a: the perceived persuasiveness of the persuasive design of the no-exposure status interface will be higher than that of the control design.
2. H2b: the perceived persuasiveness of the persuasive design of the exposure status interface will be higher than that of the control design.
3. H2c: the perceived persuasiveness of the persuasive design of the diagnosis report interface will be higher than that of the control design.

Third, research shows that adopters perceive and rate new technologies more favorably than nonadopters [71-73]. For example, Dickerson and Gentry [73] found that prior experience with other computer-related products and services played a significant role in the movement of people toward the purchase of a home computer. Hence, we hypothesized that the perceived persuasiveness of each interface will be influenced by app adoption status. In other words, given that users of COVID Alert (adopters) are familiar with and are currently using it to track their exposure, they are more likely to evaluate it favorably. Hence, we hypothesized as follows:

1. H3a: adopters are more likely to perceive the no-exposure status interface to be persuasive than nonadopters.
2. H3b: adopters are more likely to perceive the exposure status interface to be persuasive than nonadopters.
3. H3c: adopters are more likely to perceive the diagnosis report interface to be persuasive than nonadopters.

Fourth, given the hypothesized relationship between perceived persuasiveness and willingness to download the app (H1), we hypothesized that persuasive versions are more likely to be downloaded by nonadopters than control versions (H4). Some nonadopters, before the completion of the study, might have refused to download the control version of the COVID Alert app in the past for various reasons. However, with the integration of persuasive features such as self-monitoring and social learning, which provide some utilitarian benefit (monitoring of exposure levels) and a socially motivational message, we hypothesized as follows:

1. H4a: nonadopters who viewed the persuasive design of the no-exposure status interface are more likely to adopt the COVID Alert app than those who viewed the control design.
2. H4b: nonadopters who viewed the persuasive design of the exposure status interface are more likely to adopt the COVID Alert app than those who viewed the control design.
3. H4c: nonadopters who viewed the persuasive design of the diagnosis report interface are more likely to adopt the COVID Alert app than those who viewed the control design.

### Ethics Approval

This study was approved by the University of Waterloo Research Ethics Committee (ORE 42638).

## Results

In this section, we present the results based on our hypotheses. The results include the data-driven model, the mean values of perceived persuasiveness for each of the 3 interfaces, the ANOVA to uncover the main effects and interactions of factors, and the percentages of nonadopters who are willing to download the COVID Alert app from the Apple or Google store because of their awareness of it through the survey.

### Data-Driven Path Model

**Figure 5:**
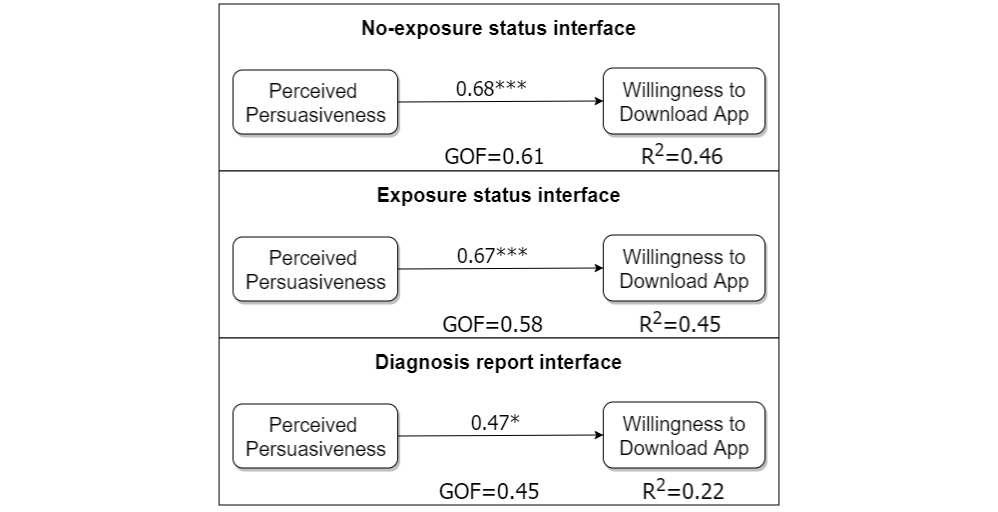
Data-driven model based on each of the 3 key user interfaces. GOF: goodness of fit. *P<.05; ***P<.001.

Figure 5 shows the data-driven models for the 3 key user interfaces. The models aim to answer the first set of hypotheses (H1a to H1c). They were built using the *partial least-squares* path modeling package in RStudio [74]. The no-exposure status interface model was built using a subset of the C1 and P1 participants (n=38) who were nonadapters, as shown in Table 2. The other 21 participants did not respond to the question on willingness to download the app. Similarly, the exposure status interface model was built using only the C2 and P2 nonadapters (n=45). Finally, the diagnosis report interface model was built using only the C3 and P3 participants (n=33). As shown in Table 1, one item was used to measure the willingness to download the app, and 3 items were used to measure perceived persuasiveness. In constructing the models, the responses *yes* and *no* to willingness to download the app were coded as *1* and *0*, respectively. All the construct items were treated as reflective indicators in the measurement models. Unlike formative indicators, which are considered the causes or drivers of the construct (ie, latent variable) that they measure, reflective indicators are considered to be caused by the construct that they measure [75]. Before analyzing the structural models, we evaluated the measurement models to ensure that the required preconditions such as indicator reliability, internal consistency reliability, convergent validity, and discriminant validity of the multiitem construct are satisfied. The outer loading metric was used to measure indicator reliability, which was >0.7 for most of the indicators that measured perceived persuasiveness in the 3 models. However, in the second model, the third indicator (*The app design is relevant to my using or continued use of the COVID Alert app*) had an outer loading value of 0.64. In the third model, the indicator was removed because its outer loading value was <0.40. The Dillion-Goldstein metric was used to assess the internal consistency reliability of perceived persuasiveness, which was also >0.7. The average variance extracted metric was used to assess the convergent validity of perceived persuasiveness, which was >0.5. Finally, the cross-loading metric was used o assess the discriminant validity of perceived persuasiveness. Its indicators loaded higher on itself than on willingness to download the app [74].

Overall, regardless of the interface, the relationship between perceived persuasiveness and willingness to download an app was statistically significant with β>0.40. We also conducted a multigroup analysis to determine the significant difference between each pair of path coefficients in the 3 submodels. The results showed no significant difference between each pair, although the path coefficients for the no-exposure status interface (β=.68; *P<*.001) and the exposure status interface (β=.67; *P<*.001) were numerically higher than those of the diagnosis report interface (β=.47; *P*=.04).

### Mean Values of Perceived Persuasiveness and RM-ANOVA

#### Overview

In this section, we address the second and third sets of hypotheses (ie, H2 and H3) by conducting a 4-factor multivariate RM-ANOVA based on the interface, app design, adoption status, and perceived persuasiveness. The results of the analysis (Table 3) show a main effect of adoption status (*F*_507,1_=28.94; *P<*.001) and an interaction between interface, adoption status, and app design (*F*_507,2_=5.90; *P=*.002). Owing to the interaction, we carried out a 2-way ANOVA taking each interface, app design, and adoption status at a time.

**Table 3:** Repeated-measure ANOVA based on interface, adoption status, app design, and perceived persuasiveness.

	Adoption status	Interface×adoption status×app design
*Df* Res^a^	507	507
*F* (*df*)	28.94 (1)	5.90 (2)
*P* value	*<*.001	.002

^a^*Df* Res: degree of freedom residual.

#### Two-Way ANOVA for Each Interface

In this section, owing to the 3-way interaction shown in Table 3, we conducted a 2-way ANOVA based on the adoption status and app design for each of the 3 interfaces.

##### No-Exposure Status Interface

**Figure 6:**
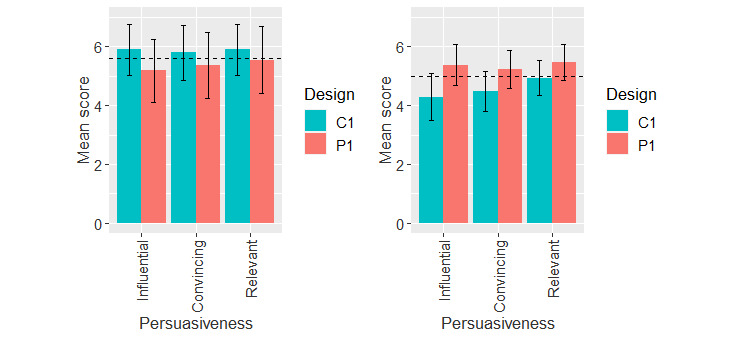
Mean ratings of perceived persuasiveness of the no-exposure interface for the COVID Alert adopters and nonadopters. Horizontal bar represents overall mean value of perceived persuasiveness. Vertical bars represent 95% CIs. C: control design; P: persuasive design.

Figure 6 shows the mean ratings of perceived persuasiveness of the no-exposure status interface for adopters and nonadopters. Overall, adopters rated the interface higher than nonadopters. As shown in Table 4, the 2-way ANOVA showed that there was a main effect of adoption status (*F*_173,1_=10.82; *P=*.001) and an interaction between adoption status and app design (*F*_173,1_=6.93; *P=*.009).

Owing to the interaction between adoption status and app design, we carried out a further 1-way ANOVA at each level of adoption status and app design as shown in Table 5. The results showed that there was a main effect of app design within the nonadopter group, with a medium effect size (*F*_112,1_=12.34; *P<*.001; η_p_^2^=0.10). The persuasive design (mean 5.37, SD 1.30) had a significantly higher mean value than the control design (mean 4.57, SD 1.19) did. Moreover, adoption status had a main effect regarding the control design, with a large effect size (*F*_79,1_=20.41; *P<*.001; η_p_^2^=0.21). The adopter group (mean 5.87, SD 1.20) rated the control design significantly higher than the nonadopter group (mean 4.57, SD 1.19).

**Table 4:** Two-way ANOVA based on adoption status and app design for the no-exposure status interface.

	Adoption status	Adoption status×app design
*Df* Res^a^	173	173
*F* (*df*)	10.82 (1)	6.93 (1)
*P* value	.001	.009

^a^*Df* Res: degree of freedom residual.

**Table 5:** Further 1-way ANOVA for the perceived persuasiveness of the no-exposure status interface at each level of adoption status and app design (small effect size: ηp2=0.01; medium effect size: ηp2=0.06; larger effect size: ηp2=0.14) [76].

			One-way ANOVA for each app design					App design effect
			C1^a^		P1^b^			
**One-way ANOVA within each adoption status**								
	Adopter	5.87		5.36		*F*_61,1_=1.05; *P*=.31		
	Nonadopter	4.57		5.37		*F*_112,1_=12.34; *P*<.001; η_p_^2^=0.10		
Adoption effect			*F*_79,1_=20.41; *P*<.001; η_p_^2^=0.21		*F*_94,1_=0.04; *P*=.84		N/A^c^	

^a^C: control design.

^b^P: persuasive design.

^c^N/A: not applicable.

##### Exposure Status Interface

**Figure 7:**
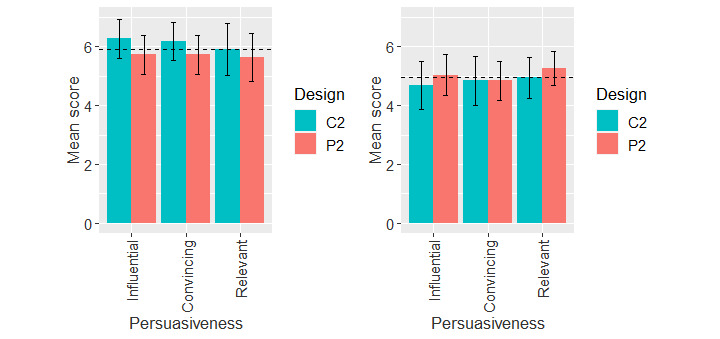
Mean scores of perceived persuasiveness of the exposure status interface for COVID Alert adopters and nonadopters. Horizontal bar represents the overall mean value of the construct for each user group. Vertical bars represent 95% CIs. C: control design; P: persuasive design.

Figure 7 shows the mean rating of the perceived persuasiveness of the exposure status interface for adopters and nonadopters. The 2-way ANOVA based on adoption status and app design (Table 6) showed that there was only a main effect of adoption status (*F*_197,1_=19.03; *P<*.001) with a medium effect size (η_p_^2^=0.09). In other words, the adopters significantly rated the perceived persuasiveness of the interface (mean 5.91, SD 1.01) higher than the nonadopters (mean 4.96, SD 1.43).

**Table 6:** Two-way ANOVA based on app design and adoption status for the exposure status interface (small effect size: ηp2=0.01; medium effect size: ηp2=0.06; larger effect size: ηp2=0.14) [76].

	Adoption status	Adoption status×app design	App design
*Df* Res^a^	197	197	197
*F* (*df*)	19.03 (1)	1.81 (1)	0.40 (1)
*P* value	<.001	0.18	0.53

^a^*Df* Res: degree of freedom residual.

##### Diagnosis Report Interface

**Figure 8:**
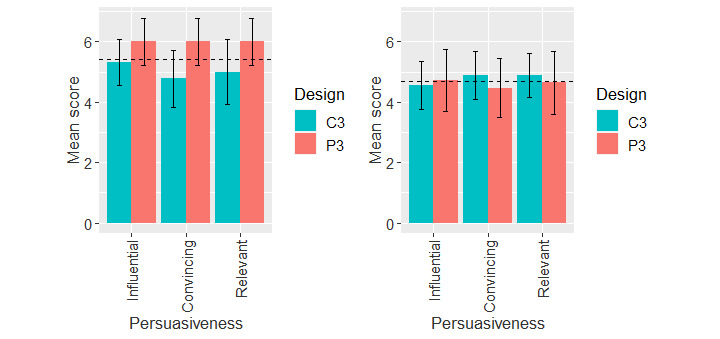
Mean ratings of perceived persuasiveness of the diagnosis report interface for COVID Alert adopters and nonadopters. Horizontal bar represents the overall mean value of the construct for each user group. Vertical bars represent 95% CIs. C: control design; P: persuasive design.

Figure 8 shows the mean rating of the perceived persuasiveness of the diagnosis report interface for the adopter and nonadopter groups. The 2-way ANOVA based on app design and adoption status (Table 7) showed that there is a main effect of adoption status (*F*_161,1_=9.51; *P=*.002) and an interaction between app design and adoption status (*F*_161,1_=4.03; *P=*.046).

Owing to the interaction between adoption status and app design, we carried out a further 1-way ANOVA at each level of each factor as shown in Table 8. The results showed that there was a main effect of app design within the adopter group (*F*_64,1_=8.00; *P=*.006), with a medium effect size (η_p_^2^=0.11). In other words, the persuasive design (mean 6.00, SD 0.97) had a significantly higher mean value for perceived persuasiveness than the control design (mean 5.03, SD 1.22). Moreover, adoption status had a main effect regarding the persuasive design, with a near large effect size (*F*_76,1_=11.10; *P=*.001; η_p_^2^=0.13). In other words, adopters (mean 6.00, SD 0.97) significantly rated the perceived persuasiveness of the persuasive design higher than that of nonadopters (mean 4.61, SD 1.84).

**Table 7:** Repeated-measure ANOVA based on app design, adoption status, and perceived persuasiveness indicator for the diagnosis report interface.

	Adoption status	App design×adoption status
*Df* Res^a^	161	161
*F* (*df*)	9.51 (1)	4.03 (1)
*P* value	.002	.046

^a^*Df* Res: degree of freedom residual.

**Table 8:** Further 1-way ANOVA for the perceived persuasiveness of the diagnosis report interface at each level of adoption status and app design (small effect size: ηp2=0.01; medium effect size: ηp2=0.06; larger effect size: ηp2=0.14) [76].

			One-way ANOVA for each app design					App design effect
			C3^a^		P3^b^			
**One-way ANOVA within each adoption status**								
	Adopter	5.03		6.00		*F*_64,1_=8.00; *P*=.006; η_p_^2^=0.11		
	Nonadopter	4.77		4.61		*F*_97,1_=0.00; *P*=.99		
Adoption effect			*F*_85,1_=0.56; *P*=.46		*F*_76,1_=11.10; *P*<.001; η_p_^2^=0.13		N/A^c^	

^a^C: control design.

^b^P: persuasive design.

#### Two-Way ANOVA for Each App Design

In this section, due to the 3-way interaction in Table 8, we conducted a 2-way ANOVA based on adoption status and interface for each of the 3 interfaces.

##### Control Design

Table 9 presents the 2-way ANOVA based on the adoption status and interface for the control design. The results show a main effect of adoption status (*F*_252,1_=20.00; *P<*.001) and an interaction between adoption status and interface (*F*_252,2_=3.45; *P=*.03).

Owing to the interaction between interface and adoption status, we carried out a further 1-way ANOVA at each level of each factor as shown in Table 10. The results show that there is a main effect of adoption status with regard to the no-exposure status interface (*F*_79,1_=20.41; *P*<.001; ηp^2^=0.21) and the exposure status interface (*F*_88,1_=21.85; *P<*.001; η_p_^2^=0.20), with a large effect size. In both interfaces, the mean value of perceived persuasiveness was significantly higher for the adopter group than for the nonadopter group. Moreover, there was a main effect of interface within adopters, (*F*_99,2_=6.33; *P=*.003), with a near large effect size (η_p_^2^=0.13), which made us carry out a further pairwise comparison. The results showed that the mean values of perceived persuasiveness for the no-exposure status interface (mean 5.87, SD 1.20) and exposure status interface (mean 6.12, SD 1.01) were significantly higher than those of the diagnosis report interface (mean 5.03, SD 1.22) at *P*=.04 and *P*=.003, respectively.

**Table 9:** Two-way ANOVA based on adoption status and interface for the control design.

	Adoption status	Interface×adoption status
*Df* Res^a^	252	252
*F* (*df*)	20.00 (1)	3.45 (2)
*P* value	*<*.001	.03

^a^*Df* Res: degree of freedom residual.

**Table 10:** Further 1-way ANOVA for the perceived persuasiveness of the control design at each level of interface and adoption status (small effect size: ηp2=0.01; medium effect size: ηp2=0.06; larger effect size: ηp2=0.14) [76].

		One-way ANOVA for each interface				Interface effect
		No-exposure status	Exposure status	Diagnosis report		
**One-way ANOVA within each adoption status**						
	Adopter	5.87	6.12	5.03	*F*_99,2_=6.33; *P*=.003; η_p_^2^=0.13	
	Nonadopter	4.57	4.82	4.78	*F*_153,2_=0.92; *P*=.40	
Adoption effect		*F*_79,1_=20.41; *P*<.001; η_p_^2^=0.21	*F*_88,1=_21.85; *P*<.001; η_p_^2^=0.20	*F*_85,1_=0.56; *P*=.46	N/A^a^	

^a^N/A: not applicable.

##### Persuasive Design

Table 11 shows the 2-way ANOVA based on adoption status and interface for persuasive design. The results show that there is a main effect of adoption status (*F*_279,1_=4.96; *P=*.03; η_p_^2^=0.03), with the mean value of perceived persuasiveness of the persuasive design being significantly higher for the adopter group (mean 5.69, SD 1.24) than for the nonadopter group (mean 5.01, SD 1.54). There is no interaction between adoption status and interface.

**Table 11:** Two-way ANOVA based on adoption status and interface for the persuasive design (small effect size: ηp2=0.01; medium effect size: ηp2=0.06; larger effect size: ηp2=0.14) [76].

	No-exposure status	Exposure status	Diagnosis report	Overall
Adopter	5.36	5.70	6.00	5.69
Nonadopter	5.37	5.05	4.61	5.01
Adoption effect	N/A^a^	N/A	N/A	*F*_279,1_=4.96; *P*=.03; η_p_^2^=0.03

^a^N/A: not applicable.

#### Two-Way ANOVA for Each Adoption Status

In this section, owing to the 3-way interaction in Table 3, we conducted a 2-way ANOVA based on app design and interface for each adoption status.

##### Adopter Group

We performed a 2-way ANOVA based on the app design and interface for the adopter group. The results showed that there was an interaction between app design and interface (*F*_189,2_=6.73; *P=*.001). Owing to the interaction, we carried out a further 1-way ANOVA at each level of app design and interface as shown in Table 12. The results show that there is a main effect of the interface with regard to the control design (*F*_99,2_=6.33; *P=*.003; η_p_^2^=0.13). There was also a main effect of app design with regard to the diagnosis report interface (*F*_64,1_=8.00; *P=*.006; η_p_^2^=0.11). Finally, there is a main effect of app design in the exposure status interface (*F*_64,1_=4.31; *P=*.04; η_p_^2^=0.06). Regarding the diagnosis report interface, the mean of perceived persuasiveness is significantly higher for the persuasive design than the control design. However, the reverse is true for the exposure status interface.

**Table 12:** Further 1-way ANOVA for adopters’ perceived persuasiveness at each level of app design and interface (small effect size: ηp2=0.01; medium effect size: ηp2=0.06; larger effect size: ηp2=0.14) [76].

			One-way ANOVA for each interface							Interface effect
			No-exposure status		Exposure status		Diagnosis report			
**One-way ANOVA within each app design**										
	Control design	5.87		6.12		5.03		*F*_99,2_=6.33; *P*=.002; η_p_^2^=0.13		
	Persuasive design	5.36		5.70		6.00		*F*_90,2_=0.98; *P*=.38		
Adoption effect			*F*_61,1_=1.05; *P*=.31		*F*_64,1_=4.3; *P*=.04; η_p_^2^=0.06		*F*_64,1_=8.00; *P*=.006; η_p_^2^=0.11		N/A^a^	

^a^N/A: not applicable.

##### Nonadopter Group

Table 13 shows a 2-way ANOVA based on app design and interface for the nonadopter group. The results showe that there is a main effect of app design (*F*_342,1_=5.62; *P=*.02; η_p_^2^=0.02), with a small effect size and persuasive design (mean 5.01, SD 1.54) having a significantly higher mean value of perceived persuasiveness than the control design (mean 4.72, SD 1.25).

**Table 13:** Two-way ANOVA based on app design and interface for the nonadopter group (small effect size: ηp2=0.01; medium effect size: ηp2=0.06; larger effect size: ηp2=0.14) [76].

	No-exposure status	Exposure status	Diagnosis report	Overall
Control design	4.56	4.82	4.77	4.72
Persuasive design	5.37	5.05	4.61	5.01
App design effect	N/A^a^	N/A	N/A	*F*_342,1_=5.62; *P*=.02; η_p_^2^=0.02

^a^N/A: not applicable.

### Nonadopters’ Willingness to Download the COVID Alert App

**Figure 9:**
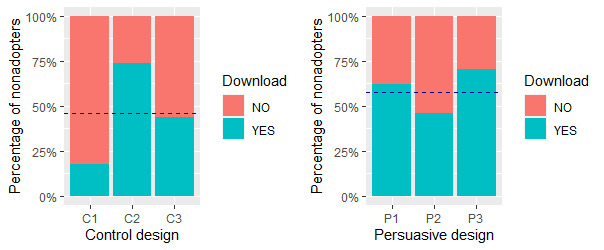
Percentages of nonadopters willing to download the COVID Alert app. Horizontal bar represents the overall percentage of nonadopters in each app design who were willing to download the app. C: control design; P: persuasive design.

This section addresses the fourth set of hypotheses (H4). Figure 9 shows the percentages of nonadopters in each of the 6 groups who were willing to download the COVID Alert app from the Apple or Google store after completing the survey. The question they responded to was *Now that I know about the COVID Alert app as the Government of Canada’s official exposure notification app, I will download it from the Apple/Google store to slow down the spread of the coronavirus*. This question was targeted only at nonadopters in the survey. Overall, the percentage of nonadopters willing to download the app from the app store was higher for the persuasive design (37/64, 58%) than for the control design (24/52, 46%).

For the no-exposure status interface, the percentage of *yes* responses was higher for P1 (13/21, 62%) than for C1 (3/17, 18%). Similarly, for the diagnosis report interface, the percentage of *yes* responses was higher for P3 (12/17, 71%) than for C3 (7/16, 44%). However, for the exposure status interface, the percentage of *yes* responses was higher for C2 (14/19, 74%) than for P2 (12/26, 46%). To investigate the statistically significant difference between each pair of interface designs (C1 vs P1, C2 vs P2, and C3 vs P3), we carried out a chi-square test as shown in Table 14. Overall, the test showed a significant difference between at least one of the pairs (χ^2^_5_=88.01; *P<*.001). Next, for the 6 user interfaces, we carried out a post hoc pairwise chi-square test using the *pairwiseNominalIndependence* function from the *rcompanion* package in R, and the Benjamini–Hochberg false discovery rate method of correction for multiple comparison errors [77]. The test showed that the persuasive and control designs for each of the 3 pairs of interfaces were significantly different (*P<*.001). We also computed the effect size (φ) based on a 2×2 contingency table for each type of interface as shown in Table 14. We used the *chisq_to_phi* function from the *effectsize* package [78] to compute the size of the effect of persuasive design on each interface. The result of the computation showed that the effect size of persuasive design for the 3 interfaces is large (φ≥0.50), with that regarding the no-exposure status interface being the highest (φ=1.01).

**Figure 10:**
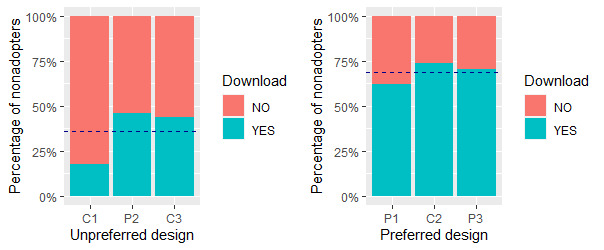
Percentages of nonadopters willing to download the COVID Alert app, with C2 and P2 switched to realize the preferred set of interfaces on the right. Horizontal bar represents the overall percentage of nonadopters in each app design who were willing to download the app. C: control design; P: persuasive design.

It is noteworthy that C2 accruing more yes responses (14/19, 74%) than P2 (12/26, 46%), coupled with the nonsignificant difference between the perceived persuasiveness of both interfaces (*P*=0.53, Table 6) indicates that the nonadopters prefer the control design of the exposure status interface over the persuasive design. Altogether, P1, C2, and P3 are preferred over C1, P2, and C3. Figure 10 shows the overall percentage of *yes* responses for each set of interfaces, with the former (39/57, 68%) exceeding the latter (22/59, 37%) by >30%.

**Table 14:** Chi-square and pairwise comparison tests for nonadopters willing to download the COVID Alert app based on Benjamini–Hochberg false discovery rate method of correction for multiple comparison errors (small effect size: φ=0.1; medium effect size: φ=0.3; larger effect size: φ=0.5) [68,78,79].

			No-exposure status interface								Exposure status interface										Diagnosis report interface										*P* value				Chi-square (*df*)
			C1^a^		P1^b^		Comparison			C2			P2			Comparison			C3				P3			Comparison									
**Willing to download the COVID Alert app**								*P*<.001; φ=1.01									*P*<.001; φ=0.57										*P*<.001; φ=0.64			<.001				88.01 (5)	
	Yes (%)	17.65		61.90					73.68			46.15						43.75				70.59													
	No (%)	82.35		38.10					26.32			53.85						56.25				29.41													
Difference (%)			−64.70		+23.80					+47.36			−7.70						−12.50				+41.48												

^a^C: control design.

^b^P: persuasive design.

## Discussion

### Principal Findings

In this section, we discuss our findings in the context of our hypotheses. For ease of reference, we summarize the key findings in Table 15. Overall, 83% (10/12) of hypotheses were fully or partially supported by the empirical data and analysis. By partial support, we mean that the hypothesis in question is only supported with regard to one of the adoption groups (adopters or nonadopters) or app designs (persuasive or control). Overall, the study reveals that adopters found the COVID Alert app, regardless of app design and use case, more persuasive than nonadopters (H3a, H3b, and H3c). Second, the study reveals that the persuasive design is more likely to be effective than the control design in motivating nonadopters to adopt exposure notification apps (H2a, H4a, and H4c) and adopters to report their COVID-19 diagnoses (H2c). In other words, our findings suggest that contact tracing apps are more likely to be effective if they are designed as persuasive technologies, particularly by incorporating self-monitoring that helps users track number of daily contacts and duration of exposure, and social learning that motivates users to report their COVID-19 diagnosis through social pressure.

**Table 15:** Summary of the validation of hypotheses.

Hypothesis (H) number	Hypothesis	Remark
H1a	The higher the perceived persuasiveness of the no-exposure status interface in the app store, the more likely users will download the COVID Alert app.	Supported
H1b	The higher the perceived persuasiveness of the exposure status interface in the app store, the more likely users will download the COVID Alert app.	Supported
H1c	The higher the perceived persuasiveness of the diagnosis report interface in the app store, the more likely users will download the COVID Alert app.	Supported
H2a	The perceived persuasiveness of the persuasive design of the no-exposure status interface will be higher than that of the control design.	Supported among nonadopters only
H2b	The perceived persuasiveness of the persuasive design of the exposure status interface will be higher than that of the control design.	Not supported
H2c	The perceived persuasiveness of the persuasive design of the diagnosis report interface will be higher than that of the control design.	Supported among adopters only
H3a	Adopters are more likely to perceive the no-exposure status interface to be persuasive than nonadopters.	Supported overall and particularly regarding the control design
H3b	Adopters are more likely to perceive the exposure status interface to be persuasive than nonadopters.	Supported overall
H3c	Adopters are more likely to perceive the diagnosis report interface to be persuasive than nonadopters.	Supported overall and particularly, regarding the persuasive design
H4a	Nonadopters who viewed the persuasive design of the no-exposure status interface are more likely to adopt the COVID Alert app than those who viewed the control design.	Supported
H4b	Nonadopters who viewed the persuasive design of the exposure status interface are more likely to adopt the COVID Alert app than those who viewed the control design.	Not supported: the reverse was the case
H4c	Nonadopters who viewed the persuasive design of the diagnosis report interface are more likely to adopt the COVID Alert app than those who viewed the control design.	Supported

### Relationship Between Perceived Persuasiveness and Willingness to Download the COVID Alert App

Our path models supported the first 3 hypotheses. Regarding each user interface, we found that the relationship between perceived persuasiveness and willingness to download the app is significant. The relationship was strongest for the no-exposure status interface (β=.68; *P<*.001), followed by the exposure status interface (β=.67; *P<*.001) and the diagnosis report interface (β=.47; *P=*.04). On the basis of the multigroup analysis, there was no statistically significant difference between each pair of path coefficients. Hence, the first set of hypotheses, *the higher the perceived persuasiveness of each interface, the more likely users will download the COVID Alert app (H1a, H1b, and H1c)*, is supported. This finding is consistent with the finding by Oyibo and Vassileva [57] in the physical activity domain. The authors found that the higher users perceive a fitness app to be persuasive, the higher their intention to use the app to motivate behavior change.

Moreover, the 3 models have an acceptably large goodness of fit (GOF), which shows how well the model fits the data. The GOF for the no-exposure and exposure status interfaces was >60%, and that of the diagnosis report interface was 38%. As stated by Hussain et al [80], a GOF for 36% is regarded as large. Moreover, perceived persuasiveness in the models regarding the no-exposure and exposure status interfaces explains at least 40% of the variance in respondents’ willingness to download the app. However, in the model for the diagnosis report interface, only 20% of the target construct was explained by perceived persuasiveness. More than 60% is regarded as a high explanation of the variance of the target construct and <30% is regarded as a low explanation [74]. Therefore, the variance in willingness to download the app explained for the no-exposure and exposure status interfaces is medium and that for the diagnosis report interface is small. These findings, which correlate with the magnitude and significance of the relationships between perceived persuasiveness and willingness to download the app (Figure 5), indicate that self-monitoring, which the no-exposure and exposure status interfaces support, is more likely to motivate nonadopters to download the app than the diagnosis reporting feature of the app. This finding may not be surprising given that notification of COVID-19 exposure and monitoring of exposure levels tend to benefit the user personally, whereas diagnosis reporting tends to benefit the community. This plausible explanation is reflected in the mean ratings of the perceived persuasiveness of the 2 interfaces by the 2 groups. For the nonadopters, the overall perceived persuasiveness of the user interfaces (Figures 5-7) is numerically higher for the no-exposure status interface (mean 5.01, SD 1.54) and the exposure status interface (mean 4.96, SD 1.43) than for the diagnosis report interface (mean 4.69, SD 1.54). Similarly, for the adopters, the perceived persuasiveness of the control interfaces (Table 10) was significantly higher for the no-exposure status interface (mean 5.87, SD 1.20) and the exposure status interface (mean 6.12, SD 1.01) than for the diagnosis report interface (mean 5.03, SD 1.22).

### App Design Effect on Perceived Persuasiveness

In this section, we discuss the effect of app design (persuasive vs control) on the perceived persuasiveness of each of the 3 user interfaces.

#### No-Exposure Status Interface

Regarding the perceived persuasiveness of the no-exposure status interface, we found an interaction between app design and adoption status (Table 4). Among the adopters, the perceived persuasiveness of the control design and that of the persuasive design did not differ significantly (*P*=.31, Table 5). However, among nonadopters, the perceived persuasiveness of the persuasive design (mean 5.37, SD 1.30) was significantly higher than that of the control design (mean 4.57, SD 1.19). The effect size of the mean difference between the 2 app designs was medium (η_p_^2^=0.10). Therefore, the fourth hypothesis (H2a), *the perceived persuasiveness of the persuasive design of the no-exposure status interface will be higher than that of the control design*, is validated for nonadopters. This finding is an indication that although the app design does not matter among adopters, it does matter among nonadopters. This implies that nonadopters are more likely to adopt the persuasive version of the no-exposure status interface (with self-monitoring features) than the control version (without self-monitoring features).

It is noteworthy that, among nonadopters, although demographic variables may confound the validation of H2a, gender is less likely to do so. This is because the gender-based distributions of the nonadopter group that evaluated the control design (C1) and that of the nonadopter group that evaluated the persuasive design (P1) were very similar. As shown in Multimedia Appendix 2, a total of 75% (12/16) of the C1 adopter group were men, and 25% (4/16) were women. Similarly, 71% (15/21) of the P1 adopter group were men, and 29% (6/21) were women. However, the percentage distributions based on age and education for the C1 and P1 nonadopters were different. For example, 24% (5/21) of the P1 nonadopter group) were aged <25 years, whereas 0% (0/15) of the C1 nonadopter group were aged <25 years. Moreover, in the P1 nonadopter group, 25% (5/20) had high school qualification, compared with only 6% (1/17) in the C1 nonadopter group. One plausible explanation for the higher percentage of participants with lower education in the P1 nonadopter group than in the C1 nonadopter group is that the former group had a higher percentage of younger participants aged <25 years. Hence, in future analyses, we hope to investigate the effect of age and education on the significant difference between the P1 and C1 nonadopter groups, which may partly account for the perception of P1 as more persuasive than C1.

#### Exposure Status Interface

Regarding the perceived persuasiveness of the exposure status interface, we did not find an effect of app design on perceived persuasiveness (Table 6). Hence, the fifth hypothesis (H2b), *the perceived persuasiveness of the persuasive design of the exposure status interface will be higher than that of the control design*, was not validated. One plausible reason why the persuasive design is not perceived as more persuasive than the control design by either adopters or nonadopters is that the information displayed on the exposure status interface is historical. In other words, the displayed information on the exposure status interface is the total sum of exposure levels over a 14-day period. This cumulative information is less transparent and unlike that of the no-exposure status interface where the displayed exposure level is for each day. Hence, the persuasive version of the no-exposure status interface, which displays daily exposure levels, was perceived as more persuasive than the control version by the nonadopter group as shown in (Table 5.

#### Diagnosis Report Interface

Regarding the perceived persuasiveness of the diagnosis report interface, we found an interaction between app design and adoption status (Table 7). Among nonadopters, the perceived persuasiveness of the persuasive design and that of the control design did not differ significantly (*P*=.99, Table 8). However, among adopters, they differed significantly (*P*=.006). Specifically, adopters perceived the persuasive design (mean 6.00, SD 0.97) to be more persuasive than the control design (mean 5.03, SD 1.22). The effect size of the mean difference between the 2 app designs was medium (η_p_^2^=0.11). Therefore, the sixth hypothesis (H2c), *the perceived persuasiveness of the persuasive design of the diagnosis report interface will be higher than that of the control design*, is validated for adopters. A plausible explanation for this finding is that having used the control design of the COVID Alert app, the adopters are likely to find the persuasive design, which incorporates social learning, more persuasive. The additional message puts the user under social pressure to follow suit, ie, join other concerned individuals who have reported their diagnosis so that exposed contacts can be notified and take the necessary safety measures to reduce the spread of the virus. The feeling of social pressure to report their COVID-19 diagnosis, fostered by the persuasive design, can be likened to the obligation and social pressure that the adopters must have felt upon the clarion call from the government and public health authorities for mass adoption to flatten the curve. However, for the nonadopters, the socially pressuring message in the persuasive design makes no significant difference compared with the control design (*P*=.99). One plausible explanation for the nonsignificant difference between both app designs among the nonadopter group is that, compared with adopters, they are less responsive to socially oriented messages, be it from the government, public health authorities, or the app. Hence, we see that the adopters in real life adopted COVID Alert owing to the clarion call from the government and public health authorities, whereas the nonadopters did not.

It is noteworthy that, among adopters, although demographic variables may confound the validation of H2c, gender and education were less likely. This is because the percentage distribution of the adopter group that evaluated the persuasive design (P3) based on gender and education and that of the adopter group that evaluated the control design (C3) look similar (Multimedia Appendix 2). For example, regarding gender, 67% (6/9) of the adopter participants who evaluated C3 were men, and 33% (3/9) were women. The same percentage distribution applies to the adopter participants who evaluated P3: 67% (8/12) were men, and 33% (4/12) were women. Similarly, regarding education, 23% (3/13) of the C3 adopters vs 22% (2/9) of the P3 adopters participants had a high school qualification, 62% (8/13) vs 56% (5/9) had a bachelor’s degree, and 15% (2/13) vs 22% (2/9) had a master’s degree. However, the percentage distributions based on age and smartphone use experience for the C3 and P3 adopter groups were different. For example, 100% (13/13) of the participants in the C3 adopter group were aged <45 years compared with 78% (7/9) in the P3 adopter group. Moreover, 85% (11/13) of the C3 adopter group had >5 years of experience, compared with 100% (8/8) of the P3 adopter group. One plausible explanation for the higher percentage of participants with more years of smartphone use experience in the P3 adopter group than in the C3 adopter group is that the former group had a higher percentage of older participants. Hence, in future analyses, we hope to uncover the effect of age and smartphone use experience on the significant difference between the P3 and C3 adopter groups, which may partly account for the perception of P3 as more persuasive than C3.

### Adoption Effect on Perceived Persuasiveness

In this section, we discuss the effect of adoption status (adopter vs nonadopter) on the perceived persuasiveness of each of the 3 user interfaces.

#### No-Exposure Status Interface

Regarding the perceived persuasiveness of the no-exposure status interface, we found an interaction between the adoption status and app design (Table 4). Regarding persuasive design (Table 5), there was no significant difference between adopters and nonadopters (*P*=.99). However, regarding the control design, there was an adoption status effect, with adopters (mean 5.87, SD 1.20) perceiving the user interface to be more persuasive than nonadopters (mean 4.57, SD 1.19). The effect size of the mean difference between the adoption statuses was large (η_p_^2^=0.21). Therefore, the seventh hypothesis (H3a), *adopters are more likely to perceive the no-exposure status interface to be persuasive than nonadopters*, is validated for the control design. A plausible explanation for this finding is that, overall, the COVID Alert adopters are more concerned with the social benefit of using contact tracing apps to curb the spread of the coronavirus than nonadopters. This explains why they are among the early adopters of the app compared with the nonadopters. Hence, it stands to reason that the adopters are more likely to perceive the COVID Alert app that they are currently using to be persuasive than the nonadopters, who are yet to adopt the app.

It is noteworthy that demographic variables such as gender and smartphone use experience may confound the validation of H3a. The reason is that the distribution of the adopter and nonadopter groups that evaluated the control design (C1) based on 3 demographic factors differs one way or the other. As shown in Multimedia Appendix 2, a total of 40% (4/10) of the C1 adopter participants were men, compared with 75% (12/16) of the C1 nonadopter group. Moreover, based on smartphone use experience, we had a higher percentage of participants with lower and higher experience in the C1 nonadopter group than in the C1 adopter group. As shown in Multimedia Appendix 2, a total of 18% (3/17) of the C1 nonadopter group had <6 years of experience and 12% (2/17) had >20 years of experience, compared with 0% (0/10) of both experience levels in the C1 adopter group. Hence, in future analyses, we hope to investigate the effect of gender and smartphone use experience on the significant difference between the C1 adopter and nonadopter groups, which may partly account for the perception of C1 by the former group as more persuasive than the latter group.

#### Exposure Status Interface

Regarding the exposure status interface, our ANOVA showed that adoption had a main effect (Table 6), with adopters perceiving the interface to be more persuasive (mean 5.91, SD 1.01) than nonadopters (mean 4.96, SD 1.43). The effect size of the mean difference between adoption status was medium (η_p_^2^=0.09). Hence, the eighth hypothesis (H3b), *adopters are more likely to perceive the exposure status interface to be persuasive than nonadopters*, is validated regardless of the app design. A plausible explanation for this finding is that, compared with the nonadopters, the adopters are more likely to be committed to the social cause of curbing the spread of the coronavirus and thus are more likely to be persuaded to use the COVID Alert app. This explains why they installed the COVID Alert app in the first place and are using it to track their exposure status (at the time of the study).

#### Diagnosis Report Interface

Regarding the diagnosis report interface (Table 7), we found an interaction between app design and adoption status regarding the perceived persuasiveness of the interface. Regarding the control design (Table 8), there was no significant difference between adopters and nonadopters (*P*=.46). However, regarding the persuasive design, there is an adoption effect, with adopters (mean 6.00, SD 0.97) perceiving the user interface to be more persuasive than nonadopters (mean 4.61, SD 1.84). The effect size of the mean difference between the 2 groups was near large (η_p_^2^=0.13). Therefore, the ninth hypothesis (H3c), *adopters are more likely to perceive the diagnosis report interface to be persuasive than nonadopters*, is validated with regard to the persuasive design. A plausible explanation for this finding is that adopters, overall, are more motivated and concerned about the social obligation to curb the spread of the coronavirus using contact tracing apps than the nonadopters, as discussed earlier in Section 5.2 Diagnosis Report Interface. In fact, not only did adopters find the persuasive design significantly more persuasive (mean 6.00, SD 0.97) than nonadopters (mean 4.61, SD 1.84) they also found it more persuasive than the control design (mean 5.03, SD 1.22). However, this is not the case for nonadopters, who did not perceive the persuasiveness of the persuasive design (mean 4.61, SD 1.84) significantly different from that of the control design (mean 4.77, SD 1.21).

It is noteworthy that apart from adoption status, demographic variables such as gender, age, education, and smartphone use experience may partly account for the significant difference between the adopter group and the nonadopter group that evaluated P3 (H3c). For example, as shown in Multimedia Appendix 2, two-thirds of the P3 adopter group were men (6/9, 67%), while one-third were men in the P3 nonadopter group (6/17, 35%). Moreover, 41% (7/17) of the P3 nonadopter group had 1 to 5 years of smartphone use experience, whereas 100% (8/8) of the participants in the P3 adopter group had >5 years of experience. Hence, in future analyses, we hope to investigate the effect of gender, smartphone use experience, and other demographic factors on the significant difference between the P3 adopter and nonadopter groups. The demographic factors may partly account for the perception of P3 by the adopter group as more persuasive than the nonadopter group. Research questions such as (1) *Are people more likely to perceive the persuasive interfaces (eg, P3) as persuasive with increase in smartphone use experience (as the percentage distribution in Multimedia Appendix 2 seems to suggest)* will be addressed and (2) *Are males more likely to perceive the persuasive interfaces (eg, P3) as persuasive than females (as the percentage distribution in Multimedia Appendix 2 seems to suggest)* will be addressed.

### Adoption Effect on Willingness to Download the COVID Alert App

Among the nonadopters, the chi-square tests regarding willingness to download the COVID Alert app show that there is an effect of user interface. This led us to carry out post hoc pairwise comparisons to uncover the effect of app design. Regarding the no-exposure status interface, the pairwise comparison shows that the size of the effect of the persuasive design is large (Table 14). This indicates that the group that viewed the persuasive design (13/21, 62%) was more willing to download the app than the group that viewed the control design (3/17, 18%). Hence, the tenth hypothesis (H4a), *nonadopters who viewed the persuasive design of the no-exposure status interface are more likely to adopt the COVID Alert app than those who viewed the control design*, is validated. This finding was replicated with regard to the diagnosis report interface. Those who viewed the persuasive design (12/17, 71%) were more willing to download the app than those who viewed the control design (7/16, 44%). Thus, the twelfth hypothesis (H4c), *nonadopters who viewed the persuasive design of the diagnosis report interface are more likely to adopt the COVID Alert app than those who viewed the control design*, is validated. The validation of H4a and H4c corroborates the findings in Table 13: among the nonadopter group, the overall perceived persuasiveness of the persuasive designs (mean 5.01, SD 1.54) is significantly higher than that of the control designs (mean 4.72, SD 1.25).

However, although the effect size tests for P1 and P3 showed that the persuasive designs were more likely to be downloaded by the participants than the control designs (C1 and C3), the reverse was true for C2 and P2. The effect size test for the exposure status interface indicated that the 11th hypothesis (H4a), *nonadopters who viewed the persuasive design of the exposure status interface (P2) are more likely to adopt the COVID Alert app than those who viewed the control design (C2)*, was not validated. Specifically, only 46% (12/26) of those who viewed the persuasive design were willing to download the app, compared with 74% (14/19) of those who viewed the control design. This finding is counterintuitive, given that the nonadopters who viewed the other 2 persuasive designs (P1 and P3) were more willing to download the app than those who viewed the control designs (C1 and C3). Although the finding is counterintuitive, it may not be far-fetched given that it aligns with the finding that among adopters (Table 12), the perceived persuasiveness of the control exposure status interface (mean 6.12, SD 1.01) is significantly higher than that of its persuasive version (mean 5.70, SD 1.02). One plausible explanation for this counterintuitive finding is the idea that the app keeps a record of the user’s total number of contacts and exposure minutes within the last 14 days (Figure 2), which, in the context of privacy, users may not like. The historical record displayed by the app may be perceived as individual surveillance [81]. Second, it has the potential to reveal the individual from whom the user contracted the virus if the total number of contacts over the 14-day rolling period was small. This may partly explain the poor performance of the persuasive version of the exposure status interface among adopters and nonadopters. Another plausible explanation for the counterintuitive finding is the relatively high hypothetical statistics presented in the P2 interface, which may be far from reality. In other words, viewing relatively high number of contacts and exposure time within the last 14 days (75 persons and 212 minutes) might have made some of them feel very uncomfortable and even doubtful. The reason for this assertion is that one would have expected the percentage of the P2 group of participants willing to download the app to be much higher given that (1) they could view the cumulative sum of their contacts and exposure minutes, which is an added value and (2) the P1 and P3 groups, who viewed the persuasive designs, were more willing to download the app than the C1 and C3 groups, respectively, who viewed the control designs. In other words, the hypothetical numbers might have been significantly higher than what the P2 group expected in a real-life setting; for example, based on their actual social distancing behavior, such as staying at and working from home. This might have caused cognitive dissonance, thereby making the P2 group doubt the accuracy of the app, which might have negatively affected their willingness to download it. In future work, we will investigate how the number of contacts and exposure time displayed in the exposure status interface influence its perceived persuasiveness and participants’ willingness to download the app.

Moreover, in future work, we will investigate the possible effects of demographic factors such as gender, age, education, and smartphone use experience on the willingness to download the app. This might help explain why the group that viewed the control design of the exposure status interface was more willing to download the app than the group that viewed the persuasive design. However, by merely inspecting the percentage demographic distribution for the C2 and P2 nonadopter groups of participants based on all 4 demographic factors, there seems to be little to no difference between the 2 groups (Multimedia Appendix 2). For example, regarding gender, 53% (10/19) of the C2 nonadopter group compared with 62% (16/26) of the P2 nonadopter group were men. Second, regarding education, 16% (3/19) of the C2 nonadopters vs 23% (6/26) of the P2 nonadopters had a high school qualification, 68% (13/19) vs 54% (14/26) had a bachelor’s degree, and 11% (2/19) vs 19% (5/26) had a master’s degree. The demographic similarities between both groups led us to the question *Apart from demographic variables, what else could possibly account for the difference between the C2 and P2 nonadopter groups in terms of their willingness to download the COVID Alert app?* The analysis of the qualitative data collected in this study and investigation of the effect of the total exposure levels displayed on the exposure status interface, in future work, can help answer this research question and gain more insights.

### Summary of Main Findings

We have shown that exposure notification apps can be designed as persuasive technologies to make them more effective in motivating behavior change. Our results revealed that exposure notification apps are more likely to be adopted and effective if they incorporate persuasive features such as self-monitoring and social learning. Our key findings can be summarized as follows:

1. Nonadopters find the persuasive design of the no-exposure interface of an exposure notification app to be more persuasive than the control design.
2. Nonadopters are more willing to download an exposure notification app with a persuasive design of the no-exposure status and diagnosis report interfaces than one with a control design.
3. Nonadopters are more willing to download an exposure notification app with a control design for the exposure status interface than one with a persuasive design.
4. Adopters are more likely to be motivated to report their COVID-19 diagnosis by the persuasive design of the diagnosis report interface than by the control design.
5. Adopters perceive the control design of the no-exposure and exposure status interfaces as more persuasive than the control design of the diagnosis report interface.
6. Adopters find an exposure notification app more persuasive than nonadopters.
7. Equipping only the no-exposure status and diagnosis report interfaces with self-monitoring and social learning, respectively, can increase adoption among nonadopters by >30%.

### Recommendations and Future Work

On the basis of the overall findings from Figure 9, a total of 58% (37/64 nonadopters) who viewed the persuasive designs were more willing to download the app from the app stores than 46% (24/52 nonadopters) who viewed the control designs. In other words, the percentage of nonadopters willing to download it from app stores increased by >10% owing to the incorporation of persuasive features into the COVID Alert app. More importantly, incorporating persuasive features into the no-exposure status interface and diagnosis report interface only has the potential to increase adoption by >30%. The exposure status interface aside, two-thirds (25/38 nonadopters) who viewed the persuasive designs were willing to download it compared with one-third (10/33 nonadopters) who viewed the control designs. This finding, together with the validation of most of the hypotheses, indicates that overall, the persuasive design of an exposure notification app is more likely to be adopted and effective than the control design. Hence, we recommend that exposure notification app sponsors work toward incorporating persuasive features such as self-monitoring and social learning into future iterations to increase adoption and user experience and make them more effective in curbing the spread of COVID-19. However, because of privacy concerns (the possibility of knowing the person from whom the user contracted the virus), displaying the total number of contacts within the last 14 days of exposure may not be advisable for the exposure status interface. In future studies, this recommendation should be investigated further. Moreover, the potential effectiveness of the other persuasive features identified in our conceptual paper (tailoring, personalization, expertise, trustworthiness, authority, praise, reward, etc) [18] should be investigated as well; for example, how would praising or rewarding the user one way or the other for uploading their one-time COVID-19 diagnosis key influence their continued use of the app or their intention to report their future diagnosis if they test positive again?

### Contributions

This study is the first to conduct research of this nature (designing contact tracing apps as persuasive technologies), using an actual exposure notification app currently being used by Canadian residents (COVID Alert app) as proof of concept. In this study, we made several contributions to knowledge regarding the persuasive design of exposure notification apps to make them more effective in curbing the spread of COVID-19. We identified and presented 3 key user interfaces (no-exposure status, exposure status, and diagnosis report). Researchers can adopt these interfaces as a basis for future research on exposure notification apps, not only for the current COVID-19 pandemic but also for other epidemics and pandemics in the future that may require exposure notification apps. Moreover, designers can work toward improving the design of exposure notification apps by incorporating persuasive features, such as self-monitoring and social learning, which we showed to be effective in the no-exposure status interface and diagnosis report interface, respectively. Finally, we showed empirically that the persuasive design of these 2 interfaces has the potential to increase adoption among nonadopters by >30%.

### Limitations

This study has limitations. The first limitation is the sample size. We only had an average of 30 participants in each of the 6 groups after data cleaning. Moreover, the participants recruited on the web (ie, on the Amazon Mechanical Turk platform) may not be representative of the entire Canadian population. For example, digital literacy and willingness to download the COVID Alert app may be higher among study participants recruited on the web [82]. This limitation may affect the generalization of the current findings to the entire Canadian population. Hence, there is a need for further research with larger sample sizes that are more representative of the Canadian population. This will help investigate how the current findings can be generalized to a larger Canadian population. Moreover, there is a need for similar research among national populations outside Canada to examine the generalizability of the findings to other countries with similar and different cultures. For example, in future, we hope to conduct a similar study among participants residing in the United States (which has an individualist culture similar to Canada’s) and Nigeria (which has a collectivist culture different from Canada’s). The second limitation of the study is the remuneration of the participants, which may have influenced their responses in some ways. The third limitation is that our findings are based on the Government of Canada’s COVID Alert app, which is only targeted at the Canadian population. Hence, there is a need for further research on country-specific apps among other national populations to investigate how the current findings generalize across different countries and cultures. The fourth limitation of this study is that we did not, in our ANOVA, investigate the main and interaction effects of important demographic variables such as gender, age, education, and smartphone use experience on the findings, although we did discuss their possible effects. The fifth limitation is that we did not investigate the entire range of persuasive strategies available from the PSD model. In addition to self-monitoring and social learning, other persuasive strategies may be instrumental in improving the persuasive design of contact tracing and exposure notification apps, with some being more likely to be effective in motivating certain health behaviors than others. Future work should address these limitations.

### Conclusions

Contact tracing and exposure notification apps may continue to be useful for a long time given the endemic potential of COVID-19 [83]. In this paper, we demonstrated that the persuasive design of an exposure notification app is more likely to be effective, using Canada’s COVID Alert as proof of concept. First, we showed that nonadopters, through self-monitoring, prefer to track their daily exposure levels (number of contacts and exposure time) in addition to knowing their exposure status. However, they are not favorable toward knowing the total number of contacts and exposure time after being notified of possible exposure to the virus. This may be due to privacy concerns, which include the possibility of knowing the individual from whom one contracted the virus, if the total number of contacts over the 14-day rolling period is small. Second, we showed that adopters are more likely to be motivated to report their COVID-19 diagnosis using a persuasive design that supports social learning (knowing how many others have reported their diagnosis) than a control design. In summary, this study indicates that equipping the no-exposure status and diagnosis report interfaces of an exposure notification app with self-monitoring and social learning, respectively, can increase the percentage of nonadopters willing to download the app by >30%. In future work, we aim to investigate how demographic variables such as age, gender, and education moderate the effectiveness of persuasive features in exposure notification app design. We also look forward to investigating the relationship between perceived persuasiveness, on one hand, and intentions to install exposure notification apps, self-isolate, and report COVID-19 diagnosis, on the other hand.

## Appendix

Multimedia Appendix 1 Administration of app interfaces to participants.

Multimedia Appendix 2 Demographics of adopters and nonadopters based on gender, age, education, and smartphone use experience.

## Abbreviations

GOF: goodness of fit
PSD: persuasive system design
RM-ANOVA: repeated-measure ANOVA
TAM: Technology Acceptance Model
