# Supplementary material for: The Effect of Persuasive Design on the Adoption of Exposure Notification Apps: Quantitative Study Based on COVID Alert
Source: JMIR Form Res. 2022 Sep 6;6(9):e34212. doi: 10.2196/34212 (PMC9450945; doi:10.2196/34212)
Supplement: Multimedia Appendix 1 [file formative_v6i9e34212_app1.docx]

# Appendix 1: Administration of App Interfaces to Participants

Table A shows the interfaces we presented to each of the six groups in our study. We randomly assigned to each participant one of the six interfaces (C1, C2, C3, P1, P2, and P3) alongside the control versions of the two other complimentary interfaces. For example, for Group 2A, we presented the persuasive version of the no-exposure status interface (P1) together with the control versions of the exposure status interface (C2) and diagnosis report interface (C3). We took this approach of presenting all three use cases of the COVID Alert App to each group of participants so that participants would have an adequate overview of the app and how it functioned prior to completing the questionnaire.

**Table A.** Functional interfaces presented to six groups of study participants. The bolded interface in each cell (e.g., P2 in cell B2) is the interface of interest for the group in question (e.g., Group B2). The unbolded interfaces (e.g., C1 and C3 in cell B2) are the complementary interfaces presented alongside the interface of interest to each group of participants

|  | Group | **A** | **B** | **C** |
| --- | --- | --- | --- | --- |
| Control Design | 1 | **C1**C2C3 | C1**C2**C3 | C1C2**C3** |
| Persuasive Design | 2 | **P1**C2C3 | C1**P2**C3 | C1C2**P3** |

The following description was provided about each of the three use cases (e.g., C1C2C3) of the COVID Alert app to each of the six groups of participants.

*Below is the Government of Canada's COVID Alert app. The app can let you and other users know of possible COVID-19 exposures before any symptoms appear. We would like you to take some time (at least two minutes) to study the three interfaces.*

*Screen 1 is the "No Exposure Notification" interface, which lets you know that you have not been exposed to COVID-19 by being close to an infected person.*

*Screen 2 is the "Exposure Notification" interface, which lets you know that you may have been exposed to COVID-19 and what to do next.*

*Screen 3 is the "Diagnosis Key Entry" interface, which allows you to enter your onetime key given to you by the public health authority if diagnosed with COVID-19.*

*When the key (in Screen 3) is entered into the app, other users, who may have come into close contact with the user who entered the key, are sent a notification (Screen 2) and provided guidance on what to do next (e.g., self-isolate or go test for COVID-19 in the event of having symptoms).*

*The app uses strong measures to protect any data it collects, and does not track a user's location or collect personally identifiable information such as name, contacts, address or health information.*

Next, we presented the bolded interface in each cell in Table A to each group of participants and asked them the questions presented in Table 2. Prior to answering the questions, participants were respectively presented with the following contextual information to set the tone for the completion of the questionnaire.

***Groups 1A and 2A:*** *Assuming you were using the Covid Alert app and you got the following exposure notification [image of no-exposure status interface ] on your mobile phone, kindly answer the following questions based on the information on the screen.*

***Groups 1B and 2B:*** *Assuming you were using the Covid Alert app and you got the following exposure notification [image of exposure status interface ] on your mobile phone, kindly answer the following questions based on the information on the screen.*

***Groups 1C and 2C:*** *Assuming you were using the app and were diagnosed with COVID-19 by public health and given a one-time key to be entered into the Covid Alert app as shown below [image of diagnosis report interface], kindly answer the following questions based on the information on the screen.*
