# Supplementary material for: The Effect of Persuasive Design on the Adoption of Exposure Notification Apps: Quantitative Study Based on COVID Alert
Source: JMIR Form Res. 2022 Sep 6;6(9):e34212. doi: 10.2196/34212 (PMC9450945; doi:10.2196/34212)
Supplement: Multimedia Appendix 2 [file formative_v6i9e34212_app2.docx]

# Appendix 2: Demographics of Adopters and Nonadopters


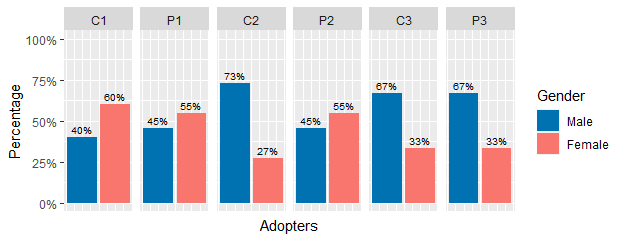


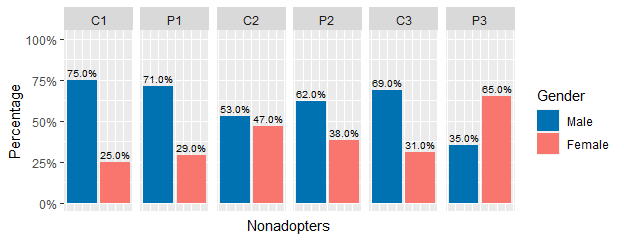

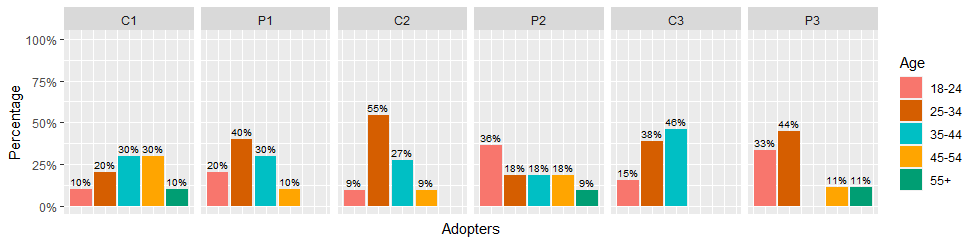


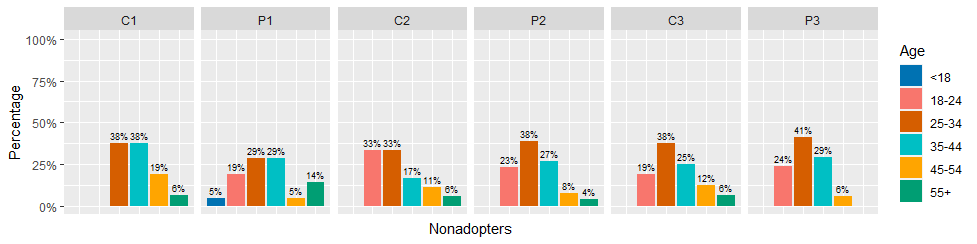


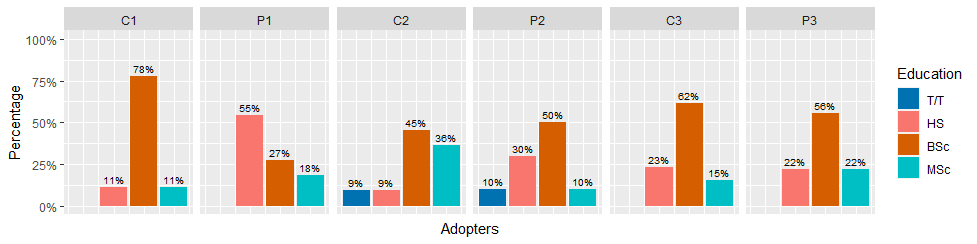


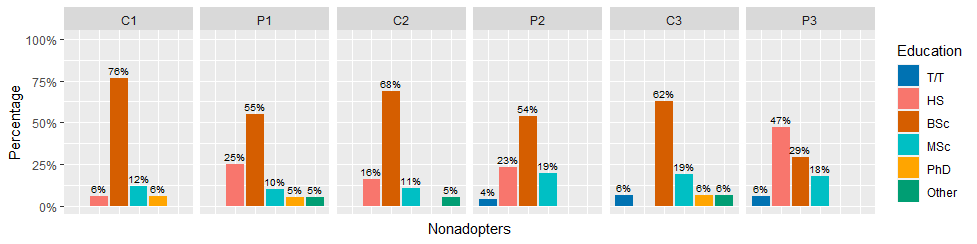


T/T: trade or technical education, HS: high school, BSc: bachelor’s degree, PhD: doctorate degree.


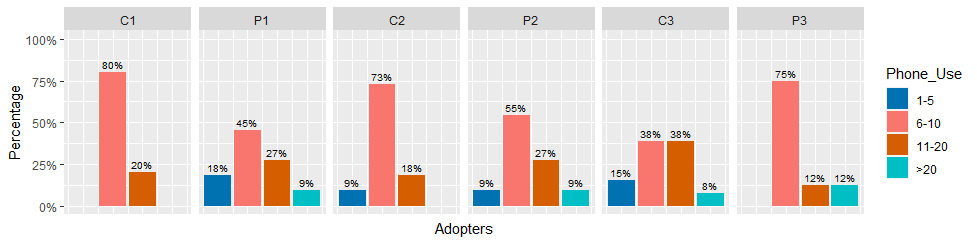


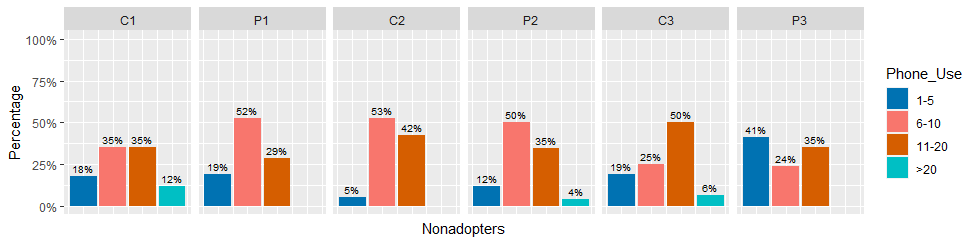


Note: Phone use is in years
